# The Potential Role of *Helicobacter pylori*-Related Mast Cell Activation in the Progression from Gastroesophageal Reflux to Barrett’s Esophagus and Esophageal Adenocarcinoma

**DOI:** 10.3390/microorganisms13081883

**Published:** 2025-08-12

**Authors:** Evangelos I. Kazakos, Efthymia Petinaki, Christos Liatsos, Ioannis S. Papanikolaou, Kyriaki Anastasiadou, Jannis Kountouras

**Affiliations:** 1 Department of Microbiology, Faculty of Medicine, School of Health Sciences, University of Thessaly, Larissa University Hospital, 41110 Larissa, Greece; ekazakos@uth.gr (E.I.K.); petinaki@uth.gr (E.P.); 2 Department of Medicine, Second Medical Clinic, Aristotle University of Thessaloniki, Ippokration Hospital, 54642 Thessaloniki, Greece; kakiaanastasiadou@yahoo.gr; 3 Department of Gastroenterology, 401 General Military Hospital of Athens, 11525 Athens, Greece; cliatsos@yahoo.com; 4 Hepatogastroenterology Unit, Second Department of Internal Medicine-Propaedeutic, Medical School, National and Kapodistrian University of Athens, 12462 Athens, Greece; ispapn@hotmail.com

**Keywords:** *Helicobacter pylori*, metabolic syndrome, mast cells, gastroesophageal reflux disease, Barrett’s esophagus, esophageal adenocarcinoma

## Abstract

*Helicobacter pylori* (*Hp*), a widespread gastric pathogen, has long been studied for its role in upper gastrointestinal disorders. While its involvement in gastritis, peptic ulcer disease, and gastric cancer is well established, its impact on esophageal diseases remains an area of ongoing investigation. Nevertheless, some data indicate that *Hp* may be involved in the pathogenesis of gastroesophageal reflux disease–Barrett’s esophagus–esophageal adenocarcinoma sequence. Similarly, the *Hp*-related mast cell activation—an essential immunological event—may also play a crucial role in the progression from gastroesophageal reflux disease to Barrett’s esophagus and esophageal adenocarcinoma. The underlying mechanisms include immune modulation, cytokine cascades, and microbial interactions that collectively shape the esophageal microenvironment. This review provides an in-depth analysis of these pathways, highlighting the potential role of *Hp*-induced, mast cell-driven inflammation in esophageal disease progression and discussing emerging therapeutic strategies.

## 1. Introduction

*Hp* infection (*Hp*-I) affects over 4.4 billion people worldwide [1] and is primarily recognized for its association with gastric pathologies. However, recent evidence suggests that *Hp* may also play a more complex role in esophageal disorders. In this context, some data indicate a potential involvement of *Hp* in gastroesophageal reflux disease (GERD)–Barrett’s esophagus (BE)–esophageal adenocarcinoma (EAC) sequence [2,3,4,5,6].

For example, a large-scale epidemiological study established that, contrary to expectations, patients hospitalized with duodenal ulcers—approximately 61,500 cases—obviously attributed to *Hp*-I burden, had a 70% increased risk of EAC [2]. Currently, no other similar large-scale studies exist. Likewise, although some studies have suggested a potential inverse correlation between Hp and BE/EAC, recent large-scale studies indicate that *Hp*-I is not inversely associated with BE. Neither the presence of erosive esophagitis, the length of BE, nor the degree of dysplasia shows a significant association with *Hp*-I [3]. Two additional studies reported that eradication of *Hp* leads to improved control of GERD symptoms and healing of esophagitis [4,5], potentially preventing its complications including BE and EAC [6].

On a molecular level, *Hp*-I induces oncogenic gastrin and other molecular alterations that contribute to the malignant progression of BE [7,8]. Specifically, *Hp*-I induces oncogenic gastrin, which plays a key role in Barrett’s oncogenic transformation by promoting cell proliferation via Janus kinase 2 (JAK2) and Akt-dependent nuclear factor-kappa B (NF-κB) activation in BE/EAC cells. It also exerts anti-apoptotic effects through upregulation of Bcl-2 and survivin and stimulates expression of cyclooxygenase-2 (COX-2), a known mitogenic and carcinogenic agent. Furthermore, *Hp* activates NF-κB, a transcription factor regulating various inflammatory genes, including COX-2, which is involved in gastrointestinal malignant cell proliferation. Prostaglandins (PGs) produced by upregulated COX-2 are implicated in BE malignant progression by sustaining chronic inflammation. These PGs promote mitogenic and anti-apoptotic effects via activation of signaling pathways such as NF-κB, Src kinase (Src), JAK2/signal transducer and activator of transcription 3 (STAT3), extracellular signal regulated kinase (ERK), mitogen-activated protein kinase (MAPK), and phosphatidylinositol 3-kinase (PI3K)/protein kinase B (Akt). In addition, *Hp*-I induces specific molecular alterations associated with BE pathophysiology, including genetic instability, E-cadherin methylation, and expression of monoclonal antibody Das-1. It also stimulates Ki-67 expression, a marker predictive of oncogenic progression in BE [8]. *Hp*-related metabolic syndrome (MetS) may further contribute to the pathophysiology of GERD–BE–EAC sequence [9,10].

Progress has been made in the management of *Hp*-I, and the roles of *Hp* and antibiotic therapies, as well as their impact on the gut microbiota, are also being considered [11]. Hypochlorhydria resulting from *Hp*-related atrophic gastritis leads to gastrointestinal dysbiosis, which—beyond its role in gastric cancer (GC)—may also promote the development of BE and EAC [12,13]. Studies of the BE biofilms have shown high levels of atypical nitrate-reducing *Campylobacter* spp. in BE tissues compared to non-BE specimens [14]. These organisms may contribute to BE development, exacerbation, and progression to EAC through chronic inflammation [13]. Therefore, *Campylobacter* species and other pathogens associated with *Hp*-induced atrophic gastritis could be etiological agents in chronic esophageal inflammation leading to EAC [13,15].

All in all, such current evidence supports a potential causal relationship between *Hp*-I and GERD complicated by BE and EAC. However, some inconclusive findings regarding the bacterium’s oncogenic potential in EAC are largely attributable to the lack of prospective cohort studies. Such studies are necessary to address novel confounding factors and clarify the role of *Hp*-I in malignant transformation within the esophagus [7].

Mast cells (MCs), as key immune regulators, play an integral role in inflammatory and oncogenic pathways. Their activation in response to *Hp*-I and MetS [16] can contribute to mucosal inflammation, epithelial remodeling, and neoplastic transformation [17,18]. Some studies have demonstrated that specific immune cell phenotypes play a pivotal role in shaping the inflammatory microenvironment in GERD, thereby facilitating progressive dysplastic changes that may lead to BE and EAC [19,20]. In this respect, the GERD–BE–EAC cascade, long attributed to chronic acid exposure and inflammation, may be significantly influenced by MC-driven responses. Understanding the interplay between *Hp*-I and MC activation is critical for elucidating the mechanisms underlying esophageal disease progression.

This review aims to provide an in-depth analysis of pathways, highlighting the potential role of *Hp*-induced, MC-mediated inflammation in esophageal disease and discussing emerging related therapeutic strategies targeting this axis.

## 2. *Hp* and MCs Activation

MCs are bone marrow-derived hematopoietic stem cells—a type of immune cell that originates from CD34^+^ and CD117^+^ pluripotent stem cells. They are involved in a wide range of physiological processes and are implicated in numerous disorders, including allergies, cardiovascular and autoimmune diseases, cancer, and even mortality [21,22]. These bone marrow-derived MCs (BMDMCs) are widely distributed in tissues, particularly at sites exposed to the external environment, such as the skin, digestive tract (including the esophagus), and respiratory tract [23,24].

In this respect, *Hp*-I promotes the migration of bone marrow-derived stem cells to the gastric mucosa, where these cells may undergo metaplastic and dysplastic transformation. This process can ultimately lead to GC through the gastric atrophy–intestinal metaplasia–dysplasia–GC sequence, as described in Coreas’ model [25,26]. Additionally, the vacuolating cytotoxin A (VacA) produced by *Hp* exerts chemotactic and activating effects on BMDMCs, prompting them to release pro-inflammatory cytokines. These cytokines contribute to both local gastric and systemic pathologies. Areas of severe inflammation, intestinal metaplasia, atrophy, and GC demonstrate increased MC densities, which are positively correlated with *Hp*-induced gastritis [27].

Beyond the GC pathway, emerging evidence suggests that *Hp* and MetS may also play a role in the pathogenesis of the GERD–BE–EAC sequence [9]. MCs have been implicated in insulin resistance (IR) and type 2 diabetes mellitus [28], parameters also associated with *Hp*-I and MetS, further contributing to the GERD–BE–EAC sequence [9].

Therefore, *Hp*-I and MetS-related activation of esophageal BMDMCs may also contribute to the GERD–BE–EAC sequence in certain ethnic populations. Eradication of *Hp* might inhibit these oncogenic processes. Thus, further research is warranted to better elucidate this field.

Specifically, *Hp*-related cytotoxin-associated gene A (CagA) and VacA are involved in the gastric inflammatory process, which can lead to gastric oncogenesis through the progression from gastric atrophy to intestinal metaplasia, dysplasia, and, ultimately, carcinoma (Coreas’ model) [29]. *Hp* disrupts the gastric epithelium through multiple virulence factors, including CagA, a protein that disrupts epithelial tight junctions and activates oncogenic signaling pathways (e.g., ERK1/2, NF-κB) via S*Hp*2 phosphorylation [30] and VacA that induces vacuole formation and modulates T-cell responses by interfering with autophagy and apoptosis. These virulence determinants contribute to immune modulation and chronic inflammation [31]. Regarding the abovementioned oncogenic signaling pathways, the available data also indicate a major role for the ERK1/2 signaling pathway in NF-κB activation in EAC [32]. Likewise, the mentioned *Hp*-related ERK1/2 signaling pathway in NF-κB activation may contribute to the pathophysiology of BE/EAC.

In particular, *Hp*-I significantly influences MC activation, a process intricately tied to chronic inflammation and disease progression in the gastrointestinal tract. MCs, in turn, play a pivotal role in orchestrating immune responses to *Hp*-I [33]. This reciprocal interaction is mediated through direct bacterial components, immune signaling pathways, and the release of MC mediators, which collectively contribute to the host–pathogen dynamic. Recent data indicate that patients infected with *Hp* had greater MC infiltration compared to uninfected individuals and that their concentration significantly correlated with the severity of inflammation [34]. *Hp* activates MC through direct and indirect mechanisms involving bacterial virulence factors, pattern recognition receptors (PRRs), and immune signaling. In this respect, *Hp*-neutrophil-activating protein stimulates MC through G-protein-coupled receptors, leading to the activation of the MAPK and PI3K/Akt signaling pathways that enhance MC survival and degranulation. This cascade results in the release of histamine and IL-6, contributing to the inflammatory milieu [35]. Remarkably, the MAPK/ERK and PI3K/AKT signaling pathways, related with *Hp*-I, are involved in the pathophysiology of both GC [36] and EAC [37].

*Hp*-related CagA protein induces IL-33 production in epithelial cells through the ERK and p38 signaling pathways, further activating MCs. IL-33 initiates a type 2 immune response by activating MC. IL-33, which is also elevated in *Hp*-infected patients [38], plays a key role in driving immune responses through MC pathways. It stimulates MC activation and proliferation, leading to degranulation and the release of preformed mediators that modulate both innate and adaptive immune cells. These mediators include IL-4 and IL-13, which promote the alternative activation of macrophages [39]. Moreover, MC release IL-2 via the IL-33/ST2 pathway, which facilitates the differentiation of CD4^+^ T cells into ICOS+ regulatory T cells while suppressing the activity of CD8+ T cells, thus fostering tumor progression [40,41]. Furthermore, binding of IL-33 to the ST2 receptor on MCs, amplifies the secretion of cytokines such as IL-6 and TNF-α, which perpetuate inflammation and support bacterial persistence [42]. It is important to note that, beyond GC [43], recent studies also demonstrate the overexpression of IL-33 during the transition from GERD to EAC, and that IL-33 promotes carcinogenesis in EAC cells through ST2 [44].

*Hp*-related VacA activates MC by promoting the secretion of TNF-α, IL-6, and IL-10 and engaging chemokine receptors to attract additional immune cells to the site of infection [45]. In turn, MC-derived cytokines such as TNF-α and IL-6 upregulate epithelial cell production of receptors that interact with VacA, enhancing its internalization and cytotoxic effects [45]. Additionally, VacA induces MC degranulation by forming pores in the cellular membrane, facilitating ion imbalance and triggering mediator release [18]. Furthermore, *Hp* lipopolysaccharide (LPS) interacts with Toll-like Receptors (TLR)4 on MCs, leading to MyD88-dependent signaling and subsequent NF-κB activation. This promotes the transcription of pro-inflammatory cytokines, including IL-1β and TNF-α [46]. *Hp* may also activate MC via multiple PRRs. TLRs 2 and 4 recognize *Hp* components and initiate downstream signaling cascades. TLR2 is overexpressed on MC in *Hp*-infected gastric tissue. NOD-like Receptors 1 and 2 (NLRs) detect peptidoglycan fragments from *Hp*, triggering inflammasome activation and IL-1β production [35,47]. Furthermore, *Hp*-I activates MC that exhibit increased GPR171 expression via the reactive oxygen metabolites (ROS)/hypoxia-inducible factor 1 alpha (HIF-1α) pathway. This in turn induces CCL2 production in MC by GPR171-ERK1/2 signaling, exacerbating gastric mucosal inflammation [34]. MCs and the mRNA/protein expression of CCL2 are significantly increased in GC, and CCL2 is associated with its development and metastasis [48].

MCs contain a diverse array of pre-synthesized and stored products with the ability to synthesize new molecules as required. The pre-synthesized substances comprise vasoactive amines like histamine, proteases like tryptase and chymase, specific cytokines like TNF-α, and growth factors such as vascular endothelial growth factor (VEGF). In this respect, for example, VEGF plays an important role in the development of *Hp*-associated GC [49], and its presence in Barrett’s mucosa is associated with an increased risk of EAC [50].

MC-derived mediators such as histamine and proteases modulate *Hp* virulence factor expression, including CagA and VacA. This dynamic feedback modulates the bacterial adaptation to the gastric microenvironment [51]. In this respect, MCs drive adaptive immune responses by promoting the following: a. T helper (Th) 2 Skewing—MCs release IL-4 and IL-13, which polarize Th cells towards a Th2 phenotype, potentially reducing the efficacy of protective Th1 responses against *Hp* [52]; b. Th17 Activation; IL-17, produced in collaboration with Th17 cells, recruits neutrophils and enhances chronic inflammation, contributing to tissue damage [17]. MC-mediated immune modulation and promotion of gastric mucosal damage may serve as key mediators in *Hp* persistence. In this respect, *Hp* exploits MC to evade immune clearance by favoring the production of regulatory cytokines such as IL-10 and TGF-β that dampen effective immune responses and the recruitment of regulatory T-cells (Tregs) to the site of infection, suppressing pro-inflammatory Th1 responses. Additionally, *Hp*-mediated MC secretion of chemokines like CCL2 attract monocytes, which differentiate into macrophages that further support chronic inflammation [16]. Sustained activation of MC leads to tryptase-related epithelial remodeling, thus accelerating the interaction of CagA with intracellular targets like S*Hp*2 and ROS-mediated epithelial injury and activation of pro-oncogenic pathways. MC-derived VEGF supports neo-vascularization, which may facilitate *Hp* adherence and survival and, thus, the establishment of chronic infection. Furthermore, histamine-induced acid secretion creates a niche that *Hp* can tolerate, aiding colonization [53].

Overall, *Hp*-related MC, beyond gastric pathology, may also exacerbate esophageal pathology through epithelial barrier disruption, nerve sensitization, and immune cell recruitment. Proteases released by MC degrade extracellular matrix components, facilitating tissue remodeling and angiogenesis [54]. The resulting chronic inflammation establishes a microenvironment conducive to metaplasia and carcinogenesis [51,52].

## 3. *Hp*, GERD, and MCs

Although the role of *Hp* in GERD remains controversial, the conventional claim that declining *Hp* prevalence has contributed to a rise in GERD requires more thorough investigation. For instance, the prevalence of *Hp*-I varies widely—from 39.9% to 84.2%—while GERD prevalence shows a lower range, from 2.5% to 51.2% [8]. These data suggest a conceivable involvement of *Hp* in the development and complications of GERD.

In this context, our findings demonstrated that *Hp*-I is common among Greek patients with GERD, including those without endoscopically confirmed disease. Furthermore, eradication of *Hp* leads to the mentioned significant symptom control and promotes healing of esophagitis [8]. Other investigators have reported comparable findings [4], highlighting the potential benefit of *Hp* eradication in preventing GERD-related complications such as BE and EAC.

Moreover, when considering the epidemiology of *Hp*-I and MetS-related GERD, various studies and meta-analyses have shown that obesity—particularly abdominal and visceral obesity—induces inflammation and contributes to the development of MetS, thus acting as an indirect risk factor for GERD [9].

**Figure 1:**
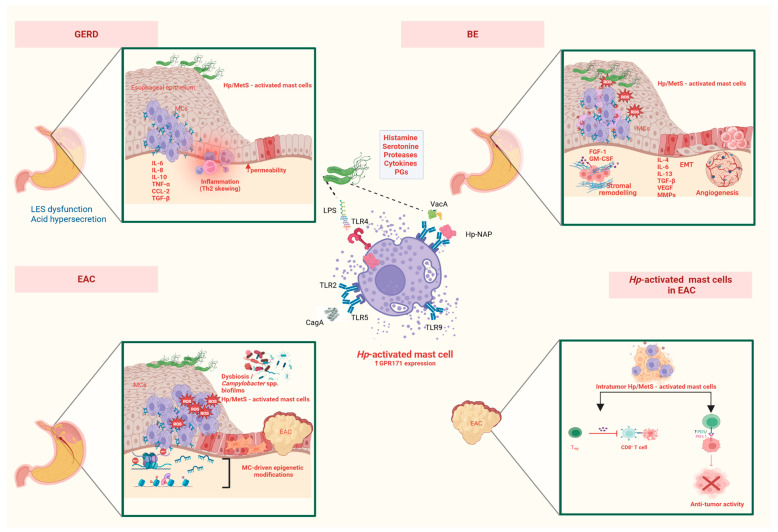
Role of *Helicobacter pylori* (*Hp*) and metabolic syndrome (MetS)-associated activated mast cells in the progression of gastroesophageal reflux disease (GERD) to Barrett’s esophagus (BE) and esophageal adenocarcinoma (EAC). CCL2, chemokine ligand 2; CagA, cytotoxin-associated gene A; EMT, epithelial-mesenchymal transition; FGF, fibroblast growth factor; GPR, g-protein receptor; GM-CSF, granulocyte-macrophage colony-stimulating factor; IL, interleukin; LPS, lipopolysaccharides; LES, lower esophageal sphincter; MMPs, matrix metalloproteinases; NAP, neutrophil-activating protein;PD-1, programmed death-ligand 1; PGs, prostaglandins; ROS, reactive oxygen metabolites; Tregs, regulatory T cells; Th, T-helper; TLR, toll-like receptor; TGF, transforming growth factor; TNF, tumor necrosis. (Created in BioRender. Kazakos, E.I. (2025) https://BioRender.com/fxncune, accessed on 3 July 2025).

Beyond such epidemiological data, GERD primarily results from lower esophageal sphincter (LES) dysfunction, which leads to acid reflux and mucosal irritation. Several lines of evidence suggest that *Hp* may contribute to GERD pathogenesis through multiple mechanisms (Figure 1), including the following:

1. Induction of mediators, cytokines, and nitric oxide that may impair LES function;
2. Direct injury to the esophageal mucosa via bacterial products;
3. Increased prostaglandin release that sensitizes afferent nerves and reduces LES pressure;
4. Enhanced gastric acidity due to *Hp*-induced gastrin stimulation, exacerbating reflux [5].

Additionally, *Hp*-induced MCs are significant effectors of the gastrointestinal–brain axis that translate stress signals into the induction of variable neurotransmitters and pro-inflammatory mediators that might contribute to gastrointestinal pathophysiology. Chronic stress stimulated by *Hp* infection results in decreased host defenses and promotes intestinal inflammation through MC-dependent mechanisms. This highlights the role of peripheral corticotropin-releasing factor receptors and MC activation in stress-related gastrointestinal disorders [55,56]. Ultimately, *Hp*-related stress appears to play a role in the onset of GERD and other gastrointestinal disorders [55], with *Hp*-induced MC activation and mediator release contributing significantly to the manifestation of major GERD symptoms [55].

Specifically, MC activation appears to be a central contributor to inflammation and tissue damage. MC-derived mediators increase epithelial permeability, induce nociceptive signaling, and amplify the cytokine milieu. TNF-α and IL-8 recruit inflammatory cells (Table 1), exacerbating tissue injury and sensory hypersensitivity [45,57]. MC influence GERD progression through the release of proteases like tryptase and chymase which degrade tight junction proteins (claudins and occludins), weakening the esophageal epithelium (Figure 1) [17]. The median number of MCs identified by tryptase staining is significantly higher in patients with erosive reflux esophagitis than in healthy controls [58]. Histamine and prostaglandins release contributes to acid hypersecretion thus aggravating acid reflux and esophageal inflammation. MC modulate the cytokine milieu through IL-8 amplifying tissue damage and TNF-α that induces NF-κB activation, a transcription factor linked to inflammation and carcinogenesis (Table 1).

The resulting inflammation heightens the risk of further esophageal damage and increases susceptibility to BE and EAC [59]. Furthermore, MC interact with the enteric nervous system, leading to neuromodulation—as histamine and tryptase activate sensory nerves (via TRPV1 and PAR2 receptors)—that exacerbates GERD symptoms [60].

Chronic acid reflux leads to epithelial injury, triggering MC infiltration and activation. *Hp* infection and MetS-associated mediators stimulate MCs) to release mediators that drive inflammation and disrupt epithelial proliferation and barrier integrity. These MCs accumulate in the subepithelial layer, secreting IL-13 and other factors that induce epigenetic changes (e.g., histone acetylation and methylation). Such alterations upregulate intestinal transcription factors, promoting metaplastic transformation. Persistent MC activity contributes to a tumor-promoting microenvironment in the esophagus. Cytokines and proteases enhance angiogenesis, facilitate immune evasion, and promote epigenetic silencing of tumor suppressor genes through DNA methylation and chromatin remodeling. MCs also modulate miRNA expression and N^6^-methyladenosine (m^6^A) RNA methylation, further amplifying oncogenic signaling pathways. 

## 4. *Hp*, ΒΕ, and MCs

Molecular pathways that drive the progression from GERD to BE involve the following: (a) Wnt/β-catenin pathway. This pathway is also associated with *Hp*-I and MetS [61,62]. It is activated by chronic inflammation, leading to epithelial proliferation and metaplasia [63]. (b) Oxidative stress-also linked to *Hp*-I and MetS [64]. Oxidative stress involves MCs producing ROS, which can induce DNA damage and mutagenesis (Table 1) [56].

BE represents an adaptive response to chronic acid exposure, characterized by the replacement of normal squamous epithelium with columnar epithelium. It is an intermediate step in the progression from GERD to EAC. This transformation is driven by sustained inflammation, with MCs playing a crucial role in cytokine secretion, epithelial proliferation, and fibrosis. The activation of the IL-6/STAT3 pathway fosters cellular survival and resistance to apoptosis, while transforming growth factor-beta (TGF-β) release promotes extracellular matrix deposition and stromal remodeling (Figure 1) [65]. These metaplastic changes create a permissive environment for further genetic and epigenetic changes that predispose to malignancy [44].

In BE, MCs stimulate epithelial proliferation and angiogenesis—key processes in metaplastic progression. The cytokines IL-13 and TNF-α contribute to an inflammatory milieu that supports cellular transformation. IL-6 and IL-13 promote epithelial proliferation and inhibit apoptosis. Furthermore, TGF-β released by MCs promotes fibrosis and angiogenesis, both hallmarks of BE (Figure 1) [56].

The median number of MCs identified by tryptase staining is significantly higher in patients with BE than in healthy controls [55]. Likewise, the proportion of Th2 effector cells (including MCs), as detected by immunohistochemical analysis, is higher in BE than in reflux esophagitis [66]. Furthermore, chronic inflammation further alters the esophageal microbiome, exacerbating the disease process [67].

MCs may also be involved in the pathophysiology of BE-associated obstructive sleep apnea (OSA). In this context, *Hp*-I may play a role in the pathogenesis of both OSA and GERD, the latter of which is also associated with OSA [68]. OSA is related with BE increased risk, due to MetS-related higher body mass index and possibly via GERD-independent mechanisms [69]. Other studies also reported a higher proportion of BE patients at higher risk for OSA [70,71].

In our series [72], we observed an association between *Hp*-I and OSA. Increased inflammatory mediator levels play a role in the pathophysiology of both OSA and MetS [71,73]. Other studies have also reported that increased *Hp* seroprevalence correlates with greater OSA severity [74].

In view of recent studies that demonstrated an association between MCs and OSA [75,76,77], *Hp*-related MCs may contribute to the pathophysiology of GERD, BE, and potentially OSA through various mechanisms. These associations warrant further investigation.

## 5. *Hp*, EAC, and MCs

Some large-scale epidemiological studies have reported that (a) patients with *Hp*-I exhibit an increased risk for the subsequent development of EAC [2], and MCs may contribute to this tumor [78], and (b) there is the absence on an increased risk of EAC after *Hp* eradication, signifying that eradication is safe from a tumor perspective [79]. Moreover, MCs may contribute to this cancer. Activated MCs are significantly increased in EAC [80]. Patients with a high-risk score for EAC also exhibit increased infiltration of activated MCs [81]. Moreover, the proportion of MCs is negatively correlated with overall survival in patients with EAC, indicating that MC infiltration is associated with a poor prognosis [81].

Apart from EAC, several studies have highlighted the role of MC in esophageal squamous carcinoma (ESC) [82], which is also associated with *Hp*-I and certain MetS-related parameters, including arterial hypertension [83,84,85,86]. A high density of MC in ESC is associated with progression and low postoperative survival, potentially involving mechanisms such as E2F targets, epithelial–mesenchymal transition, G2/M checkpoints, mitotic spindle dynamics, and the TNF-α/NF-κB inflammatory pathway [87].

More specifically, regarding EAC pathophysiology, hypochloridria accompanying *Hp*-induced atrophic gastritis may trigger early dysbiotic events involving the predominance of the mentioned *Campylobacter* spp. biofilms (mainly *Campylobacter consisus*) with a contributory role in the GERD–BE–EAC cascade, primarily through increased expression of the cancerogenic IL-18 (Figure 1) [14], microbiome alteration, and cytolethal distending toxin-mediated genotoxic effects [88]. Furthermore, *Campylobacter* spp.-specific signatures correlate with high levels of active MCs in metaplastic tissues, thereby contributing to BE progression [89]. Compared to controls, patients progressing through the EAC cascade exhibit a higher prevalence and abundance of emerging *Campylobacter* species [88].

Mechanistic insights into the molecular events that drive transition from BE to EAC reveal that *Hp*-associated MC activity, in the context of a Th2 humoral profile shift, may facilitate oncogenesis through oxidative DNA damage and the promotion of angiogenesis [65]. In addition to cytokine signaling, MCs contribute to an oxidative microenvironment by recruiting neutrophils and producing ROS (Figure 1), which further promotes epigenetic silencing through upregulation of DNA methyltransferases [90,91]. Upon activation by bacterial components such as *Hp* VacA and LPS, MCs secrete a cascade of pro-inflammatory cytokines including TNF-α, IL-6, and IL-13, all of which potentiate ROS generation either directly or by recruiting neutrophils and macrophages. These interactions enhance NADPH oxidase activity and mitochondrial dysfunction, leading to the accumulation of hydrogen peroxide and superoxide anions in the local tissue microenvironment [45,92]. In the esophagus, oxidative damage is most evident in the metaplastic and dysplastic stages of BE. MC-derived mediators, in concert with *Hp*-induced inflammation, create a redox-rich milieu that fosters carcinogenesis in distal esophageal epithelium [91]. This is consistent with observations of elevated oxidative DNA lesions such as 8-hydroxy-2′-deoxyguanosine (8-OHdG) in both the gastric and esophageal mucosa in *Hp*-infected patients [93]. MC-derived ROS also modulate key signaling pathways that promote tumorigenesis. Oxidative stress activates NF-κB, HIF-1α, and STAT3—transcription factors that drive survival, angiogenesis, and immune evasion and accelerates p53 mutations related to impaired aging and DNA repair capacity, pivotal in EAC progression (Table 1). Moreover, MC-derived tryptase enhances, via protease-activated receptor 2 (PAR-2), inflammatory cell recruitment and ROS-dependent apoptosis resistance in epithelial cells [94]. Signaling via PAR-2 elicits activation of the MAPK phosphorylation family and contributes to a pro-malignant transcriptional shift coupled with increased oncogenic protein synthesis and the production of pro-angiogenic factors, such as VEGF, IL-8, IL-6, granulocyte-macrophage colony-stimulating factor (GM-CSF), which is also linked to *Hp*-I [95,96], and macrophage colony-stimulating factor [97]. Furthermore, chronic gastritis and microbial imbalance induced by *Hp* can promote GERD, which exposes the esophageal epithelium to bile acids and gastric acid—exacerbating the oxidative cascade, that is central to the inflammation–metaplasia–dysplasia sequence of EAC development [98,99].

MCs contribute to the angiogenic switch via secretion of the mentioned VEGF, matrix metalloproteinases (MMPs), TNF-α, and IL-8, TNF-α, and IL-8, facilitating neovascularization and tumor expansion [82]. In esophageal tumors, MC infiltration positively correlates with microvessel density, highlighting their spatial and functional relevance in tumor-associated angiogenesis and poor prognosis [81]. This has been documented in ESC and is increasingly recognized in EAC, where MC-derived VEGF-A and MMP-9 enable both endothelial cell recruitment and extracellular matrix remodeling, paving the way for invasive tumor growth [21,100]. This paracrine signaling loop supports a microenvironment conducive to angiogenesis, fibrosis, and tumor progression. Forma et al., further elaborated on the angiogenic repertoire of MCs, noting their ability to secrete both VEGF-A and CXCL8 in response to hypoxia and inflammatory stimuli-conditions common in the *Hp*-colonized gastric and esophageal mucosa [101]. In this respect, *Hp*-I may enhance MC-driven angiogenesis in EAC. *Hp* has been shown to increase the expression of angiogenic markers such as VEGF-A, ANGPT1/2, and TNF-α in gastric tissues (Table 1) [102]. Although direct studies in EAC are limited, similar inflammatory processes in the gastric mucosa may extend to the esophagus, especially in the context of *Hp*-induced reflux esophagitis or metaplasia. Furthermore, extracellular vesicles from gastric epithelial cells infected with *Hp* can carry pro-angiogenic signals such as miRNAs and cytokines that promote angiogenic gene expression in adjacent stromal cells, including MCs and endothelial progenitors [103]. This paracrine signaling loop supports a microenvironment conducive to angiogenesis, fibrosis, and tumor progression. While direct evidence linking *Hp* to angiogenesis in EAC via MCs is still emerging, the mechanistic parallels in gastric carcinogenesis and the shared inflammatory milieu support this association. Targeting MC activity or their angiogenic mediators may, thus, offer a viable therapeutic strategy in EAC, especially in *Hp*-associated or inflammation-prone individuals. Additionally, studies in GC show that MCs often co-localize with T-regulatory cells and are enriched in tumor regions with active neovascularization, suggesting a role in both immunomodulation and vascular expansion. Although most mechanistic studies focus on gastric malignancies, the underlying biology is relevant to EAC due to shared inflammatory and metaplastic pathways.

Chronic *Hp* colonization triggers sustained inflammatory signaling, including the activation of NF-κB, secretion of pro-inflammatory cytokines such as IL-6 and TNF-α and IL-15, which in turn mobilize immune cells like dendritic cells, macrophages, and notably MCs [104]. Histological data from EAC tissues consistently report increased clusters of degranulated MCs localized in proximity to dysplastic and metaplastic esophageal epithelium, often adjacent to invasive tumor fronts [105]. This spatial correlation suggests a mechanistic role in facilitating the transition from chronic inflammation to neoplastic transformation and underscores the direct involvement of MC-derived mediators to the stromal remodeling and extracellular matrix degradation essential for tumor invasion. Microbiome analyses further suggest that MC activity is shaped by the host–microbiota interface. Decreased diversity in gut and esophageal microbial communities, often driven by *Hp*, reflux-mediated dysbiosis, MetS-related central obesity, administration proton-pump inhibitors and antibiotics, can enhance MC sensitization through microbial-associated molecular patterns (MAMPs) [106,107]. These interactions, coupled with gastric reflux that damages the epithelium, thus exposing TLRs to MAMPs, stimulate further TLR pathways, specifically TLR4 and augment local Th2 immunity, fostering fibrosis and epithelial plasticity [108]. In this respect, EAC may be characterized as a “microbiome-modulated malignancy,” where immune mediators such as MCs shape the tumor microenvironment by interpreting microbial cues into inflammatory and oncogenic signals [109].

MCs play an ambiguous role by suppressing antitumor T cell-mediated immunity (Figure 1). MCs have been found to express immune checkpoint molecules, including programmed death-ligand 1 (PD-L1), especially under the influence of *Hp*-induced inflammation. In GC models, increased MC density correlates with enhanced TNF-α/NF-κB-mediated PD-L1 expression in the tumor microenvironment and diminished CD8^+^ T-cell infiltration, creating an immune-suppressive niche that limits cytotoxic T-cell responses and facilitates immune escape in *Hp*-exposed esophageal epithelium and promoting tumor growth [106,110]. The immunosuppressive function of MC is further amplified by their secretion of TGF-α, IL-10, and TNF-α cytokines known to polarize T-cells toward a regulatory phenotype or induce exhaustion in effector populations. *Hp*-induced MC activation triggers these mediators through recognition of bacterial components via TLRs and PRRs, leading to a skewed Th1/Th2 balance and impaired dendritic cell–T cell cross-talk [111]. *Hp*-infected antigen-presenting cells can downregulate T cell proliferation and IFN-γ production, highlighting a mechanism that may be further sustained by MC-derived prostaglandins and histamine, which suppress T-cell receptor signaling and migration [112]. In a clinical context, MC-driven immunosuppression has been implicated in resistance to immune checkpoint inhibitor (ICI) therapy. A recent study reported poorer outcomes in patients with advanced GC and *Hp* positivity receiving PD-1/PD-L1 inhibitors, potentially due to an immunologically “cold” tumor phenotype dominated by suppressive myeloid and MC populations [113]. *Hp*-driven MC activity may lead to silencing of tumor suppressor genes, most notably CDKN2A (p16), through both epigenetic and immune-mediated mechanisms. CDKN2A encodes the p16INK4a protein, a cyclin-dependent kinase inhibitor essential for regulating G1-S phase progression and preventing uncontrolled cell proliferation. Loss of p16 expression—via genetic deletion or epigenetic silencing—is one of the earliest and most frequent molecular events in the BE–EAC sequence [114]. Promoter methylation of CDKN2A has been reported in early metaplastic and dysplastic lesions [115], and recent studies have shown that CDKN2A deletion synergizes with KRAS activation to accelerate neoplastic transformation in the esophageal epithelium [116]. Transformation of murine MC via constitutive KIT activation was associated with Cdkn2a/Arf loss, suggesting an intrinsic ability of MCs to influence tumor suppressor pathways [117]. Activated MCs release cytokines such as IL-6, TNF-α, and IL-13, which are known to promote transcriptional repression and DNA methylation at tumor suppressor loci via STAT3 and NF-κB signaling cascades (Table 1) [118,119]. In EAC, chronic inflammatory states correlate with sustained methylation of CDKN2A, silencing E-cadherin and promoting epithelial–mesenchymal transition, further reinforcing tumor progression. *Hp*-induced inflammatory cascade, amplified by MCs, further suppress CD8^+^ T cell responses via PD-L1 expression and IL-10 signaling, creating an immune-privileged environment where tumor suppressor gene inactivation—such as that of CDKN2A—can proceed unchecked [106,113]. Together, these insights delineate a complex but cohesive pathophysiological model wherein *Hp*-driven MC activation act as a catalyst promoting the epigenetic silencing of antitumor genes like p16 in EAC. This occurs through a synergy of cytokine secretion, redox imbalance, immune suppression, and chronic inflammation.

The progression from GERD to BE and EAC is orchestrated by a complex interplay of immune cells, cytokines, and molecular pathways. MCs serve as pivotal players, modulating inflammation, epithelial remodeling, and tumor progression. Understanding these mechanisms provides opportunities for therapeutic intervention targeting specific immune pathways and microbiome modulation to disrupt this pathological cascade (Table 2).

## 6. *Hp*-Mediated MC Activation and Epithelial–Mesenchymal Transition

Epithelial–mesenchymal transition (EMT) is a critical process in which epithelial cells lose their polarity and adhesion properties, transitioning to a mesenchymal phenotype with enhanced motility and invasiveness [120]. EMT plays a pivotal role in fibrosis, inflammation, and tumor progression in *Hp*-associated diseases, including its involvement in the pathogenesis of GERD and its sequelae, namely BE and EAC (Figure 1).

In EAC, especially cases preceded by *Hp*-I, MC activation has emerged as a crucial link between microbiome-driven mucosal inflammation, EMT, and neoplastic transformation. Chronic *Hp* colonization triggers sustained inflammatory signaling, including the activation of NF-κB, secretion of pro-inflammatory cytokines such as IL-6 and TNF-α and IL-15, which in turn mobilize immune cells like dendritic cells, macrophages, and notably, MC [104]. In this context, MCs chronically activated by persistent microbial stimuli like *Hp*, lead to aberrant cytokine secretion (e.g., IL-6, IL-13, TNF-α) and release a spectrum of bioactive mediators—histamine, proteases (tryptase, chymase), prostaglandins, and leukotrienes—that alter epithelial integrity, disrupt tight junctions, and support the recruitment of Th2-polarized immune responses, compounding EMT and fibrotic remodeling. MCs amplify a *Hp*-triggered cascade involving IL-8 and other pro-inflammatory cytokines, perpetuating epithelial damage [17]. Of particular interest is the influence of MC-derived IL-4 and IL-13, which have been shown to prime epithelial cells for EMT via TGF-β and STAT6 signaling cascades, promoting fibroblast-like characteristics and migration potential [57]. These signals contribute to the reprogramming of epithelial cells into mesenchymal phenotypes via upregulation of EMT drivers, such as Snail, Twist, and Zinc Finger E-Box Binding Homeobox (ZEB) 1 [104,121], that enhance invasiveness and resistance to apoptosis—hallmarks of EAC progression [122]. In the esophagus, this contributes to mucosal injury and the establishment of a pro-oncogenic niche, conducive to BE and progression to EAC. Notably, MC-secreted MMPs and fibrogenic cytokines, including IL13, drive fibrosis and facilitate cell migration, thereby creating conduits for invasive tumor growth and angiogenesis, key features of EMT-mediated metastasis [123].

In the context of GERD, chronic acid exposure induces inflammation and oxidative stress in esophageal epithelial cells, triggering EMT through pathways involving TGF-β, NF-κB, and Wnt/β-catenin signaling [93]. This transition contributes to fibrosis, strictures, and the metaplastic transformation of squamous epithelium into intestinal-type epithelium, characteristic of BE [124].

Further along this pathological sequence, BE cells undergoing EMT exhibit increased stemness, invasion, and resistance to apoptosis, predisposing them to dysplasia and progression to EAC [125]. Markers of EMT, such as Snail, Twist, and ZEB1, have been correlated with increased cancer risk and poor prognosis in EAC patients [126]. MCs, as active modulators of the inflammatory microenvironment, significantly contribute to EMT through the release of bioactive mediators and interactions with the microbiome. Mechanisms of MC-mediated EMT include the following: (a) Cytokine-Mediated Pathways that directly trigger EMT-associated signaling in epithelial cells, such as TGF-β that initiates Smad-dependent signaling pathways, thus repressing epithelial markers (e.g., E-cadherin) and inducing mesenchymal markers like vimentin and fibronectin (Table 2) [127]. MC-derived TGF-β exacerbates *Hp*-induced EMT [52], and IL-6 activates the Jak2/STAT3 signaling pathway that upregulates EMT transcription factors, such as the mentioned Snail, Twist 1, and ZEB 1 and 2, reinforcing the pro-migratory phenotype of epithelial cells and enhancing cell motility and invasiveness, especially in hypoxic or ROS-rich microenvironments [128]. (b) Protease-Mediated Pathways that remodel the extracellular matrix (ECM). MC-derived tryptase and chymase upregulate MMP-9 expression in epithelial cells, whereas MC-secreted granzyme B, responsible for the release of proangiogenic factors, such as FGF-1 and the mentioned GMCSF from the ECM, enhances ECM degradation and facilitates cellular invasion and weakening of the basement membrane, thereby promoting epithelial cell migration and tumor invasion [129,130]. (c) ROS generated by MCs contribute to EMT by inducing oxidative damage that disrupts epithelial integrity and by activating NF-κB and HIF-1α (hypoxia-inducible factor 1-alpha), both associated with EMT and tumor progression [131]. Respectively, *Hp* virulence factors amplify MC-mediated EMT. CagA disrupts cell polarity and activates β-catenin signaling. MC-derived cytokines and proteases enhance the localization of β-catenin in the nucleus, promoting EMT [35], whereas VacA synergizes with MC-derived inflammatory mediators to suppress epithelial junctional proteins, facilitating mesenchymal transformation [132]. Furthermore, MC-driven inflammatory responses play a central role to the microbiome–EMT axis [133]. *Hp*-induced dysbiosis results in the accumulation of pro-inflammatory microbial metabolites, including LPS, whereas refluxed bile acids such as taurodeoxycholic acid further select for LPS-producing bacteria, amplifying inflammation. This synergy coupled with dietary influences disrupts gut microbial communities, promoting the enzymatic conversion of primary to secondary bile acids including deoxycholic acid and lithocholic acid that act as potent mediators of epithelial stress. TGR5 receptor-mediated TLR4 activation on MCs modulates MC activity and epithelial gene expression, which enhances EMT and metastasis by targeting VEGFR2 and sustaining chronic inflammation [59,134].

Short chain fatty acids (SCFAs) like butyrate, which typically inhibit EMT by stabilizing epithelial junctions, are depleted in dysbiosis, removing a key regulatory mechanism that would otherwise limit MC activation and inflammation [135]. *Hp*-I is also correlated with elevated production of SCFAs, such as acetate and propionate—microbial metabolites essential to gut health—while concurrently reducing populations of butyrate-producing bacteria, leading to persistent chronic inflammation and metabolic disruptions [136,137]. Pathogenic microbes in a dysbiotic microbiome also stimulate MC degranulation, increasing the release of EMT-inducing mediators like IL-6, TNF-α, and prostaglandins and activate MCs via microbial PAMPs (e.g., LPS) that bind TLRs, perpetuating chronic inflammation and promoting EMT (Table 2) [51]. In this respect, dysbiotic microbial communities composed of Gram-negative anaerobes and microaerophiles—abundant in BE—like *Veillonella*, *Prevotella*, *Fusobacterium* [132], and *Hp* biofilms, interact with MCs to remodel the extracellular matrix composition, via ROS, miRNAs, and cytokine networks, making it conducive to cellular migration and invasion and facilitate persistent inflammation and oxidative stress, driving EMT [105]. *Hp* biofilms provide a platform for delivering signaling molecules like LPS and quorum-sensing autoinducers via outer membrane vesicles (OMVs), which activate the TLR4/MD2 receptor complex—a known MC activator-enhancing pro-inflammatory response [138]. OMVs carry virulence factors that promote microbial coaggregation, enhance immune evasion, and stabilize biofilms [139]. *Hp* OMVs have been implicated in protecting the bacterium from ROS generating during the immune response, thereby enhancing its survival [140]. Moreover, *Hp*-related OMVs contain various virulence factors and may amplify the bacterium’s overall pathogenic potential. These OMVs can also induce autophagy, relying on the nucleotide-binding oligomerization domain-1-receptor interacting serine/threonine kinase 2 signaling pathway, which is critical for autophagy induction and IL-8 production [141]. Additionally, *Hp*-OMVs stimulate autophagosome formation, independent of VacA [139], and through IL-8 production and NF-κB activation, may contribute to gastric pathologies.

The failure of antibiotic eradication in *Hp*-I infection is partly due to the bacterium’s ability to hide within host cells, thereby evading immune responses. Increasing evidence suggests that macroautophagy/autophagy plays a significant role in the pathogenesis of *Hp*-associated gastric disorders [142]. Different *Hp* strains exhibit variations in their capacity to release OMVs and form biofilms. Biofilm formation enables *Hp* to survive antibiotic exposure and promotes bacterial colonization and persistence in the stomach [143]. Moreover, biofilm formation can influence the effectiveness of antibiotics in eradicating susceptible bacterial strains [144]. Efflux pumps, which are proteinaceous transporters, actively expel antimicrobial agents from the bacterial interior, lowering intracellular drug concentrations. Since efflux pumps underwrite to both antimicrobial resistance and biofilm formation, a comprehensive understanding of their mechanisms may be crucial for developing new therapeutic strategies against *Hp* [143].

MCs are pivotal in promoting EMT through cytokine release, protease activity, and oxidative stress. This process is compounded by dysbiosis, which enhances MC activation and EMT-promoting signals via microbial metabolites and *Hp* biofilm interactions (Table 2).

## 7. Therapeutic Implications of *Hp*-Driven MC Activation in the GERD–BE–EAC Sequence: Targeting the MC–EMT–Microbiome Axis

*Hp*-induced chronic inflammation activates EMT, leading to epithelial disruption and malignant transformation [104,145]. Molecular tools now allow modulation of EMT via transcriptional, epigenetic, and microenvironmental interventions. Nanoparticles and exosomes engineered with EMT-targeting ligands (e.g., anti-vimentin antibodies) enhance targeted delivery to MC-infiltrated tissues, attenuating transcription of both upstream and downstream EMT mediators, improving bioavailability and minimizing off-targets [146,147].

MC stabilizers demonstrating promising effects in patients with functional gastrointestinal disorders (e.g., cromolyn sodium, nedocromil, lodoxamide, ketotifen) inhibit degranulation and may mitigate *Hp*-driven injury [22,35,148,149]. Tyrosine kinase inhibitors (e.g., imatinib, masitinib) targeting the c-Kit receptor, tryptase inhibitors (e.g., gabexate mesylate, nafamostat mesylate, tranilast), and the JAK1/JAK2 inhibitor ruxolitinib also show potential and are currently undergoing clinical evaluation targeting MC-related pathologies [150,151,152,153].

Targeting IL-33/ST2, PI3K/Akt/mTOR, and PD-1/PD-L1 pathways can reduce MC activation and restore immune surveillance [106,154,155,156]. Additional immunomodulators like tocilizumab, infliximab, and CCL2/CCR2 blockers can attenuate *Hp*-driven pro-inflammatory cascades and tumor–stroma interactions [157,158].

Dual administration of proton pump inhibitors with TGF-β or IL-6/STAT3 inhibitors reduces both bacterial colonization and EMT induction [159]. Agents like XAV939 that destabilize β-catenin reduce its transcriptional activity suppressing Wnt/β-catenin-driven EMT progression and, thus, EMT-driven tumorigenesis exacerbated by *Hp*-I [145]. PAR1 is also involved in promoting cancer invasiveness and dissemination, making it a potential target for therapeutic strategies [160,161]. MMP inhibitors like marimastat and ROS scavengers like N-acetylcysteine attenuate extracellular matrix degradation and MC-derived oxidative stress, respectively, known contributors to DNA damage and EMT in *Hp*-exposed tissue [162,163,164].

Restoration of microRNAs (miRNAs), such as the miR-200 family, via synthetic mimics and the use of siRNAs targeting EMT drivers (e.g., Snail, Twist) present promising strategies to maintain epithelial identity in gastric epithelial cells. Delivery via nanoparticles or liposomes enhances tissue-specific uptake in inflamed gastric mucosa [165,166,167].

In combination therapies, the integration of antibiotics for *Hp* eradication with MC-targeted immunomodulators offers synergistic benefits. Eradication regimens co-administered with cromolyn sodium or anti-cytokine agents may enhance therapeutic outcomes, reducing inflammation and preventing the evolution of BE or EAC. Cromolyn is a selective and strong drug in inhibiting the proliferation of cancer cells [168].

Epigenetic therapies (e.g., vorinostat, azacytidine, JQ1), alone or in combination with anti-inflammatory agents, suppress EMT transcription and restore epithelial gene expression [162,169,170,171]. Furthermore, fusions of dCas9 with demethylases can reprogram hypermethylated promoters of epithelial markers, offering precision control over gene expression to restore epithelial homeostasis [170]. Similarly, CRISPR/Cas9 gene editing allows the knockout of EMT genes, such as Snail and Twist, which has been shown to halt EMT initiation and potentially reverse fibrotic remodeling in *Hp*-induced models [172,173].

Modulation of the gut microbiome has also emerged as a critical adjunct in mucosal immune priming restoring homeostasis and preventing dysbiosis-induced miRNA-targeted epigenetic regulation, which promotes marked intratumorally infiltration of activated MCs [121]. Probiotics have demonstrated promising effects in patients with functional gastrointestinal disorders, such as GERD [149], and its potential complications including BE and EAC [174]. Probiotics such as *Lactobacillus rhamnosus* and *Bacillus* have demonstrated the ability to inhibit MC degranulation by down-regulating the expression of the high-affinity IgE receptor (FcεRI) and histamine H4 receptor (H4R), both of which are critical for antigen-induced MC activation and histamine-mediated signaling [175,176]. These probiotics also engage TLR2-dependent mechanisms on MC surfaces to modulate intracellular signaling cascades, including MyD88-dependent inhibition of NFκB activation, thus suppressing the transcription of pro-inflammatory mediators such as TNF-α and IL-6 [177]. Interestingly, emerging data suggest that *Bifidobacterium* may play a protective role in preventing colorectal cancer progression by modulating MC activity [178].

In animal models, administration of probiotics (*Lactobacillus plantarum*) resulted in a notable decrease in the relative abundance of *Clostridium sensu stricto 1*, associated with impaired intestinal permeability and MC activation [179]. Moreover, both in vitro and in vivo studies have shown that certain lactobacilli, including *Limosilactobacillus fermentum*, attenuate MC degranulation by significantly reducing the release of β-hexosaminidase, a well-established marker of MC activation [180]. Suggested mechanisms may involve stabilization of MC membranes or interference with calcium mobilization pathways necessary for granule exocytosis [181,182]. Furthermore, probiotics including *Lactobacillus* and *Bifidobacterium* have been shown to compete with *Hp* for epithelial binding, reduce inflammation, and rebalance microbial ecology. Fecal microbiota transplantation (FMT) presents an advanced strategy to reverse *Hp*-driven dysbiosis and suppress MC hyperactivity through microbial–host crosstalk, a hypothesis supported by preliminary clinical observations. FMT offers promising opportunities to restore microbial balance and enhance treatment effectiveness, potentially leading to better outcomes for patients with esophageal cancer. Incorporating microbiome-targeted strategies into existing treatment approaches may improve the management of esophageal cancer, reduce side effects, and increase patient survival rates [183].

### Limitations

This study has certain limitations. Some data indicate that *Hp* may be involved in the pathophysiology of GERD, and eradication of *Hp* has also been associated with improved symptom control and healing of esophagitis [4,5]. Moreover, GERD patients with concurrent *Hp*-I have shown reduced symptom rebound following *Hp* eradication [184]. However, other studies report an inverse association—indicating that *Hp* infection may reduce GERD risk, and its eradication may actually increase that risk [185]. Therefore, more research is needed to clarify the role of *Hp* in GERD development and symptom recurrence.

Additionally, while we discussed the involvement of *Hp* and MCs in the GERD–BE–EAC sequence, it remains unclear whether these mechanisms are driven primarily by gastric or esophageal MCs, or both. Further investigation is warranted.

Lastly, targeting the MC–EMT–microbiome axis is currently supported mainly by in vitro and experimental data, with limited human evidence. More clinical studies are necessary to validate these findings.

## 8. Conclusions

The complex interplay between *Hp* and MC encompasses not only immune modulation and epithelial damage but also microbial ecology and stromal remodeling. Understanding the molecular and cellular pathways that mediate this relationship reveals novel therapeutic targets across the GERD–BE–EAC spectrum. Interventions that combine microbial eradication with immune stabilization, oxidative stress mitigation, and EMT suppression hold transformative potential. This integrative therapeutic approach may significantly improve outcomes in patients with *Hp*-associated esophageal diseases and redefine current treatment paradigms. Therefore, strategic future directions are essential to effectively mitigate the global burden associated with *Hp*-related MC activation in the pathophysiology of the GERD–BE–EAC sequence.

## Figures and Tables

**Table 1:** Summary of the Immune Cascade in GERD–BE–EAC.

Disease Stage	Key Immune Cells	Major Cytokines/Pathways	Key Outcomes
GERD	MCs, neutrophils, macrophages	IL-6, TNF-α, NF-κB	Chronic inflammation, epithelial damage
BE	MCs, Th17-cells, macrophages	IL-17, TGF-β, Wnt/β-catenin	Metaplasia, oxidative stress
EAC	MCs, TAMs, Tregs, MDSCs	VEGF, STAT3, PI3K/AKT, NF-κB	Angiogenesis, immune evasion, EMT

BE, Barrett’s esophagus; EMT, epithelial–mesenchymal transition; EAC, esophageal adenocarcinoma; GERD, gastroesophageal reflux disease; IL, interleukin; MCs, mast cells; MDSCs; myeloid derived suppressor cells; ΝF-κΒ, nuclear factor-kappaB; PI3K/AKT; phosphoinositide 3-kinase/protein kinase B; Tregs, regulatory T cells; STAT3, signal transducer and activator of transcription 3; Th, T-helper; TGF, transforming growth factor; TAMS, tumor associated macrophages; TNF, tumor necrosis factor; VEGF, vascular endothelial growth factor.

**Table 2:** Key Molecular Pathways in Mast Cell-Driven epithelial–mesenchymal transition (EMT) and Microbiome Interactions.

Pathway	MC Role	Microbiome Contribution
**TGF-β/Smad** **Pathway**	Secretes TGF-β to initiate Smad-dependent EMT	Dysbiosis enhances MC activity through LPS
**STAT3 Pathway**	IL-6 release activates STAT3 and EMT transcription	Loss of SCFAs removes STAT3 inhibition
**NF-κB Signaling**	Drives chronic inflammation and oxidative stress	Microbial PAMPs activate NF-κB in MCs

EMT, epithelial–mesenchymal transition; IL, interleukin; LPS, lipopolysaccharides; MCs, mast cells; ΝF-κΒ, nuclear factor-kappaB; PAMPs, pathogen-associated molecular patterns; SCFAs, short-chain fatty acids; STAT3, signal transducer and activator of transcription 3; SMAD, small mother against decapentaplegic homolog; TGF, transforming growth factor.

## Data Availability

No new data were created or analyzed in this study. Data sharing is not applicable to this article.
